# Editorial: Microbiology of deep-sea carbon cycling

**DOI:** 10.3389/fmicb.2023.1236593

**Published:** 2023-07-03

**Authors:** Rulong Liu, Yong Wang, Gordon Webster

**Affiliations:** ^1^Shanghai Engineering Research Center of Hadal Science and Technology, College of Marine Sciences, Shanghai Ocean University, Shanghai, China; ^2^Institute for Ocean Engineering, Shenzhen International Graduate School, Tsinghua University, Shenzhen, China; ^3^Microbiomes, Microbes and Informatics Group, School of Biosciences, Cardiff University, Cardiff, United Kingdom

**Keywords:** deep-sea, carbon cycling, microorganisms, diversity, metabolic activity

The ocean is a vast carbon sink and mediates global carbon cycling, essential for mitigating climate change. The deep-sea pelagic and sub-seafloor environments represent the largest microbial habitats on Earth and are key sites for organic matter remineralization and storage in the biosphere. Moreover, diverse unique and extreme habitats, e.g., seamounts, trenches, cold seeps, and hydrothermal vents exist in the deep sea, developing special and active microbial communities and metabolic processes that significantly impact global carbon cycling. It is therefore important to understand the diversity, activity and metabolism of deep-sea microorganisms, particularly their mechanisms for utilization and transformation of organic matter, and the environmental factors affecting these processes. The main aim of this Research Topic is to collect recent work focusing on the diversity and metabolic activities of microorganisms in different deep-sea habitats, in order to understand the microorganisms that drive carbon cycling in the deep ocean.

Marine sediments harbor diverse physicochemical properties that regulate the assemblages of microorganisms. However, it is unclear how variations in sediment physicochemical properties impact microorganisms on a global scale. Bradley et al. investigated patterns in the distribution of microbial cells, organic carbon, and the amounts of power used by microorganisms in global sediments. They found that trends in cell abundance, particulate organic carbon storage and degradation, and microbial power utilization are mainly structured by depositional settings and redox conditions, rather than sediment depth and age. Sediments deposited on continental shelves and margins are predominantly anoxic and contain active microbial cells that decline in power utilization in deeper and older settings. Conversely, microorganisms in abyssal sediments use consistently low amounts of power across large gradients in sediment depth and age. Overall, the study demonstrated broad global-scale connections between depositional settings and activity of deep biosphere microorganisms.

Zhang et al. compared the composition and functions of the microbial communities in sediments from deep-sea seamounts, trenches and cold seeps in the Pacific Ocean, via amplicon sequencing and metagenomic analysis. They demonstrated that the microbes in deep-sea sediments were diverse and were functionally different (in terms of biogeochemical cycling) from each other in the seamount, trench, and cold seep ecosystems. These results help improve the understanding of the composition, diversity and function of microbial communities in deep-sea environments.

Deep-sea seeps are extreme environments with high hydrostatic pressure, yet the seep systems have a great impact on global carbon cycling through discharge of methane and petroleum hydrocarbons. Webster et al. characterized the microbial diversity, geochemistry and methanogenic activities of prokaryotic communities in seven Gulf of Cádiz mud volcanoes. They concluded marked differences between the microbial biogeochemistry of mud volcano sediments and deep-sea control sediments. They found that methanogenic activities from methyl compounds, especially methylamine, within the top two meters of sediment were much higher than with the substrates H_2_/CO_2_ or acetate. The potential archaea responsible for the methanogenic metabolisms were explored and sediment enrichments were dominated by *Methanococcoides* methanogens.

Lyu et al. investigated the potential and activities of deep-sea microorganisms for alkane degradation in the sediments of cold seep areas. They enriched five oil-degrading consortia from sediments collected from the Haima cold seep areas of the South China Sea, and further isolated seven efficient alkane-degrading bacteria belonging to *Acinetobacter, Alcanivorax, Kangiella, Limimaricola, Marinobacter, Flavobacterium*, and *Paracoccus*. The degradation rates of these bacteria were the highest in alkanes with medium chains. This study provides insights into the community structures, and oil-degrading activity of the bacterial inhabitants in the Haima cold seep areas, South China Sea, and offers bacterial resources for cultivation of candidates with oil bioremediation application potential.

## Author contributions

RL wrote the draft. YW and GW revised and provided essential comments on the article. All authors have proofread and approved it for publication.

